# Application of a microcomputer system in an infra-red laboratory

**DOI:** 10.1155/S146392468300005X

**Published:** 1983

**Authors:** M. J. Adams, I. Black

**Affiliations:** The Macaulay Institute for Soil Research Craigiebuckler Aberdeen AB9 2QJ UK


Journal of Automatic Chemistry, Volume 5, Number 1 (January–March 1983), pages 9–13

### Application of a microcomputer system in an infra-red laboratory

**M. J. Adams and I. Black**

*The Macaulay Institute for Soil Research, Craigiebuckler, Aberdeen AB9 2QJ, UK*

### Introduction

The increasing availability of modern microcomputer systems and their advantages is now appreciated by analytical instrument manufacturers and laboratory staff. Offering powerful computing facilities for moderate cost, the microcomputer has the major virtue of functioning readily in conversational mode, rather than the batch-processing mode associated with large, conventional mainframe units. Newton has recently discussed the features offered by some of the more common microcomputers [1] and, in general, the system can be expected to comprise a microprocessor, a high-level language, input-output (I/O) facilities, random-access memory (RAM), and a permanent storage medium such as magnetic tape or disc. Many computer manufacturers and suppliers, appreciating the demand for these systems in laboratories, have made available a wide range of low-cost interfacing and data-communication modules. One such general-purpose system has been described by Broadbridge and Heslop [2]; in many cases the interfacing of a microcomputer to a modern analytical instrument is a simple task. However, the preparation of adequate and suitable software (programs) for the efficient use of the system can be a more serious problem. Although several companies sell specialized software for scientific use, most microcomputer programming is done in-house by the user and the ease of learning and using the BASIC language certainly aids the non-specialist in program development. Nevertheless, it is still true that the major cost of efficiently employing a microcomputer system in a laboratory is the software for the particular application.

Analytical instrument manufacturers have been quick to realize the advantages offered by microprocessor-controlled instruments and several now offer dedicated microcomputers for instrument control and subsequent data processing [3 and 4]. Whilst the use of these systems relieves the user from having to develop the necessary software, such dedicated instruments are, in general, expensive and inflexible in that they are difficult to modify for uses other than their original purpose.

Infra-red spectroscopy, since its inception as a routine analytical technique, has made increasing use of computer methods for data analysis. It is to be expected, therefore, that microcomputers will become more common in the infra-red laboratory. In this paper, the use of a typical microcomputer for instrument control, data logging and data analysis is described. Interfacing and software development problems are discussed, and the results compared with instrument manufacturers' dedicated computer facilities.

### Instrumentation

The infra-red spectrometers and computing system are illustrated schematically in figure 1. Essentially, the laboratory contains a research quality, dispersive spectrometer (Perkin-Elmer's Model 580B) and a Fourier spectrometer (Beckman-RIIC's Model FS720). Located away from the infra-red laboratory, but linked with the microcomputer, is a mainframe computer (Data General's Eclipse).

The Apple II microcomputer is typical of the 'personal' computers now available. It is controlled by an eight-bit, type 6502 microprocessor operating at a clock frequency of 1 MHz and having a 16-bit parallel address bus allowing direct adressing to 64 k bytes of memory. The monochrome monitor display employed is standard, as supplied, with 24 lines of text, at 40 characters/line, or graphics at 280 points by 160 points with four lines of text below the graphics display. Text and graphics are memory-mapped via RAM. A 12 in monitor (18 MHz video bandwidth) provides higher quality definition of the graphics display than a conventional television receiver, which has typically a 5–6 MHz video bandwidth. Permanent program and data storage is achieved with twin $5\frac{1}{4}$ in floppy disc units (Apple disc operating system, DOS 3·3), each of which is capable of storing in excess of 120 k bytes of data. Communication and data transfer between the spectrometers, mainframe computer and the microcomputer is achieved via standard interface cards connected directly to the I/O ports of the computer. As well as on-line data-logging from the spectrometers, spectral data can also be transferred to the microcomputer with the aid of a graphics digitizing tablet. Spectral data from the computer can be plotted on the 580B spectrometer or on a flat-bed plotter (Watanabe's Digi-Plot), and a small, thermal printer is available for listings, providing a permanent copy of disc catalogues, etc.

Although large-capacity (1–10 M byte) disc-storage systems are becoming more common for microcomputers, they usually cost more than the computer system itself, so bulk data storage on a mainframe computer is being investigated here as an alternative. Data storage on mainframe computers has the advantages associated with the operation of the larger units, for example efficient data-file management and copying facilities.

The 580B dispersive infra-red spectrometer is a double-beam, ratio-recording instrument containing its own microprocessor unit and can be supplied complete with a serial, RS232C, communications interface for computer control and data logging. The usual controller is the Perkin-Elmer Data Station, a dedicated microcomputer system with ready-to-use software for the spectrometer [5 and 6]. The spectrometer is capable of operation within the range $4000\,cm^{-1}$ to $180\,cm^{-1}$, spectra being recorded on an integral strip-chart recorder.

The Michelson Fourier spectrometer provides a useful extension to the working range of the more conventional dispersive instruments and generally operates in the range $600\,cm^{-1}$ to $40\,cm^{-1}$, depending on the beam splitter employed, with a typical resolution of $2{\cdot}5\,cm^{-1}$. Before the microcomputer system was available, the data from the interferometer was recorded on punched paper-tape which was taken to the mainframe computer for data-processing and spectrum plotting.

### Interfacing

The major interfacing links between the units in the system are shown in figure 1. The 580B and the mainframe computer share

© 1983 The Macaulay Institute for Soil Research

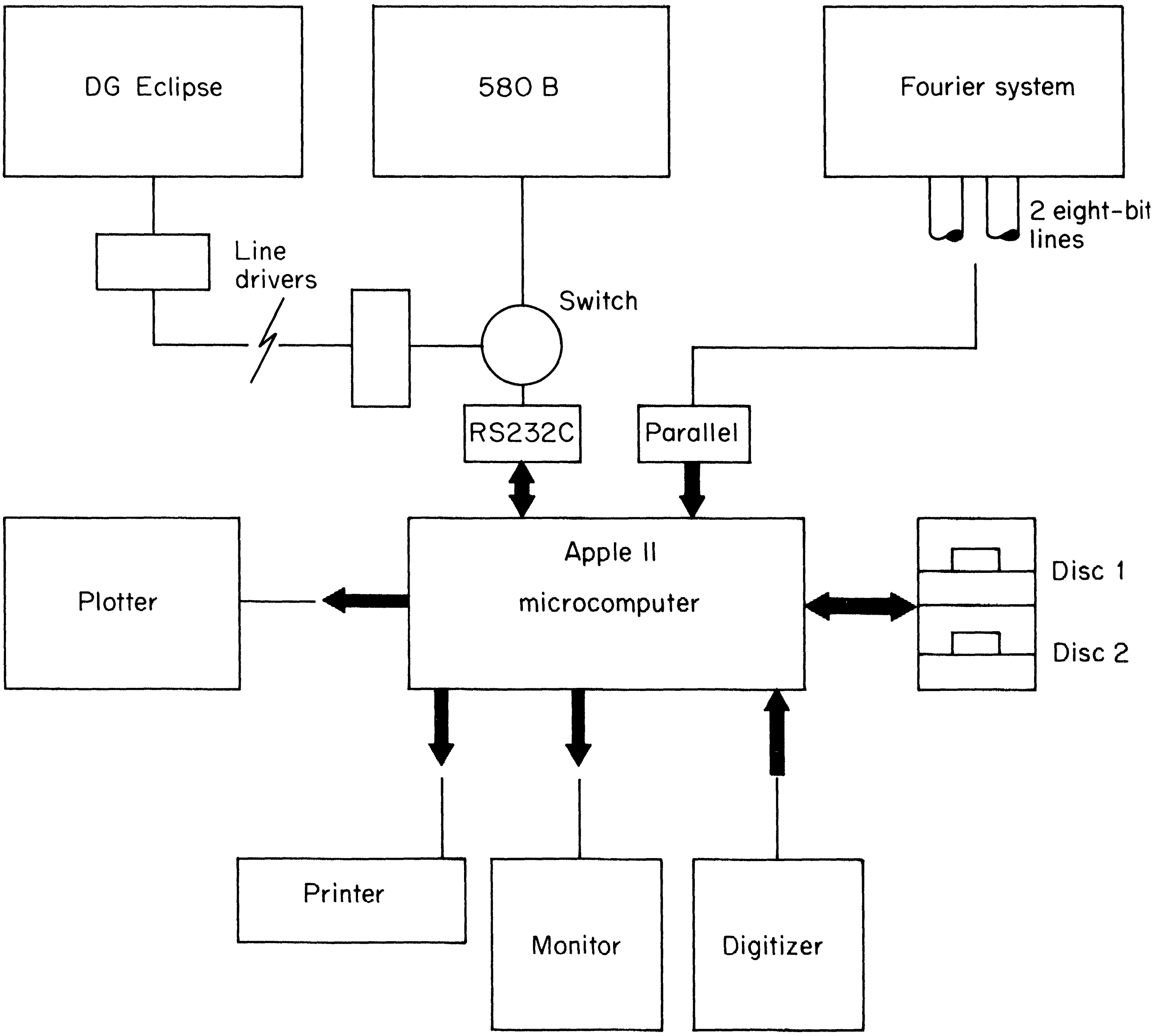

*Figure 1. Schematic representation of the microcomputer system and the interfacing links to the dispersive spectrometer (580B), the Fourier spectrometer, the mainframe computer (Eclipse) and peripheral devices.*

a single RS232C serial interface in the microcomputer, the Apple II treating both as intelligent remote terminals. Selection between the computer and the spectrometer is achieved by an external switch. In both cases a shielded three-wire link serves for data and control signal transmission and reception. A Model 7710A asynchronous serial interface (California Computer Systems) is fitted to the microcomputer and operated at 600 baud. The link to the spectrometer from the switch is direct, and via a pair of line-drivers (Nolton Communications, type 1171) to the mainframe computer. The relatively slow baud rate, selected by a switch on the interface card, was chosen to provide minimum data loss in transmission to and from the Eclipse mainframe whilst still providing efficient control of the 580B spectrometer. It should be noted that, at data transmission rates below 600 baud, the spectrometer interface can overwrite data within its processing system, resulting in loss of spectral data.

The standard electronic processing system with the Fourier spectrometer is the FS300 unit. This consists of the instrument's power-supplies, detector amplifiers, a Moiré amplifier system for monitoring the displacement of the moving mirror, an analogue-to-digital converter and a matrix and paper punch drive module. The latter was discarded and the output to the microcomputer taken directly from the 16-bit BCD analogue-to-digital converter unit. A twin, eight-bit parallel interface (California Computer Systems, Model 7720) in the microcomputer monitors the data, as two eight-bit digits, from the spectrometer. To avoid timing problems associated with parallel data transmission, the computer-interferometer link has been limited to a length of approximately 1 m.

### Software development

The infra-red applications software has been prepared in modular form to operate in conversational mode from displayed menus. All programs are contained on a single disc in Drive-1, and operate with, or on, spectral data on a second disc in Drive-2. The master menu, with the selection options offered

(table 1), operates as a turnkey system requiring no computing knowledge from the user. The use of separate program modules for the distinct functions allows for ease of program development and modification. The only constraint necessary for the individual modules to be compatible is that spectral data formatting be consistent. Unless stated otherwise, all programs are written in BASIC. Whilst higher-level languages, for example Pascal and Fortran, are available for the Apple II microcomputer, their use in this machine can limit some I/O options.

Complex and large data-handling programs, as used in this application, require extensive use of the microcomputer memory-map in the development of software. A typical memory-map for the Apple II microcomputer, and as used in the MANIPULATION module, is illustrated in figure 2.

### Program modules

### *580B*

Coates [5] and Coates and Geary [6] have described the use of the 580B spectrometer with the Perkin Elmer Data Station, and Chipperfield and Kirby [7] have used a Digico M16V minicomputer for control and data processing. The communications interface of the 580B is accessed via a serial, RS232C port in the spectrometer and a series of software commands are recognized by the interface for control of the instrument. All communication between the spectrometer and microcomputer is handled by machine-coded routines, to overcome the relatively slow operating speed of BASIC. The operator command options for the 580B microcomputer module (see table 2) consist of commands communicating directly with the spectrometer and disc- or file-based commands.

All commands to the spectrometer consist of a two-letter mnemonic code preceded by a $ character, automatically supplied by the computer. Certain commands also require a numeric parameter. Thus, $GO 4000 causes the monchromator unit to advance to 4000 $cm^{-1}$. Transmission data are digitized in the range 0 to 20000, corresponding to 0% and 100% transmission respectively. Calibration, scanning with data-logging and the replotting of computer-recorded data are initiated by commands which are automatically compiled within the computer and require minimum operator input. Although a wide range of scanning modes and sample digitizing intervals can be selected for use with the spectrometer, it is preferred that all spectra, where permissible, be recorded with a pre-selected set of

*Table 1. Modules.*

Master menu

- 580B

  - Discs

  - Spectrometer

- Fourier system

  - Logging

  - Processing

  - Plotting

- Manipulation

  - Files

  - Arithmetic

- Plotter

- Terminal

  - Eclipse

  - 580B

- File development

- Quit — out to BASIC

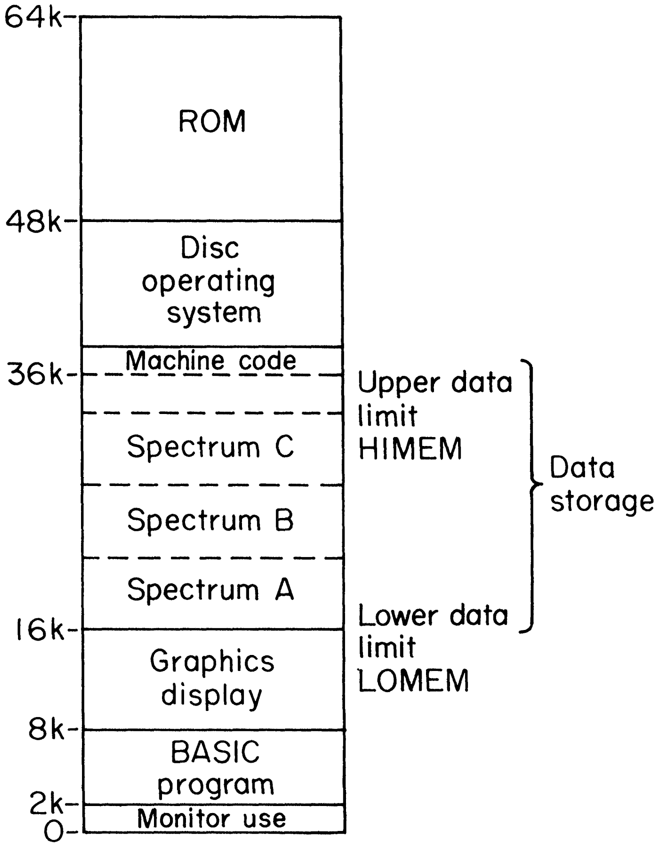

*Figure 2. Memory-map for the Apple II microcomputer with 48k bytes RAM. The data-storage area (including three spectra) is located between the address limits of the memory-mapped graphics display and the disc operating system. A small area, typically 2k bytes, is reserved for machine-coded routines.*

standard conditions. This procedure provides for greater ease of use and prevents mismatch problems in comparing and manipulating spectral data. The majority of spectra, therefore, are recorded with a sampling interval of 2 $cm^{-1}$ in the region 4000 $cm^{-1}$ and 2000 $cm^{-1}$ and of 1 $cm^{-1}$ from 2000 $cm^{-1}$ to 180 $cm^{-1}$; a total of 2821 points is thus used to represent a complete spectrum.

Replotting of the spectral data on the spectrometer requires each data point to be reformatted to two six-bit ASCII characters for transmission to the spectrometer.

The CONTROL routine allows complete operator control of the spectrometer by means of the full range of commands recognized by the spectrometer processing system.

The remaining routines within this module are concerned with disc operations. Digitized spectral data can be saved to, or loaded from the data disc and any spectrum on a disc can be viewed, in graphics mode, prior to its replotting on the 580B spectrometer.

### *Fourier system*

This module consists of a data-logging program, from the FS 720/300 spectrometer system, and provision for the subsequent transformation, processing and plotting of the data (see table 3). The twin eight-bit parallel lines between the spectrometer and microcomputer are unidirectional, to the computer, with no control commands to the spectrometer being necessary. A machine-coded, interface routine monitors data transmission. The double-sided interferogram, selected by the operator to be based on 1024 or 2048 points, is recorded at a fixed sampling interval of 8 $\mu$m. The untransformed data are converted from 16-bit BCD to binary before storage.

The transformation routine in the microcomputer is similar to that supplied by Beckman-RIIC and originally used with a mainframe computer. An analogous program for a single-sided interferogram has been published by Jones [8]. Background correction of transformed spectra is achieved by ratioing a sample against a pre-recorded, transformed, blank spectrum. Printed output of the spectral data is achieved with the flat-bed plotter and the scaling is compatible with that used for spectra obtained from the dispersive spectrometer. An example of the output is shown in figure 3.

### *Manipulation*

This program module (table 4) is used for the examination and arithmetical manipulation of spectral data recorded from the 580B dispersive spectrometer, and is similar in operation to that described by Coates [5] and Jones [9]. Although written as a single program, for convenience it can be considered in two parts: file management and active manipulation. File-management commands do not modify the spectral data but allow spectra to be retrieved from, or passed to, disc storage. The data can also be transferred within the microcomputer memory system. Up to three spectra can be held in RAM simultaneously (figure 2), and viewed, between operator-specified wave-number limits, on the video monitor. The arithmetic manipulation routines modify data and include procedures for absorbance/transmission conversion, zero offsetting, flattening a base-line (by a quadratic fit procedure), curve smoothing using the Savitsky-Golay procedures [10], summing spectra and determining the difference spectrum from two samples. An example of the use of the latter function is illustrated in figure 4. All commands require only a single key entry, the first letter of the command, for selection.

### *Plotter*

The Digi-Plot flat-bed plotter interfaces directly to the Apple II via a parallel communication line. The PLOTTER module is employed in producing printed output of spectra in a wide variety of format scales, and works in conjunction with the

*Table 2. The 580B module.*

| | |
|---|---|
| 580B | 1. CALIBRATION |
| | 2. SCAN (with data logging) |
| | 3. PLOT (on the spectrometer) |
| | 4. CONTROL (of the spectrometer) |
| | 5. LOAD (spectrum from disc) |
| | 6. SAVE (spectrum to disc) |
| | 7. VIEW (spectrum on disc) |
| | 8. CATALOGUE (contents of disc) |
| | 9. QUIT (to master menu) |

*Table 3. The Fourier system module.*

| | |
|---|---|
| Fourier system | 1. DATA LOGGING |
| | 2. TRANSFORMATION |
| | 3. RATIO |
| | 4. PLOT |
| | 5. QUIT (to master menu) |

*Table 4. The manipulation module.*

| | | |
|---|---|---|
| Manipulation | A(DDS) | Addition of a constant |
| | B(ASE-LINE) | Shift spectrum to the base-line |
| | D(IFF) | Difference between two spectra |
| | E(RASE) | Clear graphics screen |
| | F(LAT) | Flatten background |
| | G(ET) | Load spectrum from disc |
| | K(LIP) | Erase selected spectral data |
| | L(IST) | Catalogue data disc |
| | M(ULT) | Product of spectrum and a constant |
| | N(OISE) | Smoothing routines |
| | Q(UIT) | Exit program, to master menu |
| | R(ECORD) | List operations performed |
| | S(UMS) | Addition of two spectra |
| | T(AAT) | Transmission/absorbance conversion |
| | V(IEW) | Graphics display of selected spectrum |
| | X(CHANGE) | Data transfer within microcomputer |
| | Z(OOM) | Expansion of graphics display |

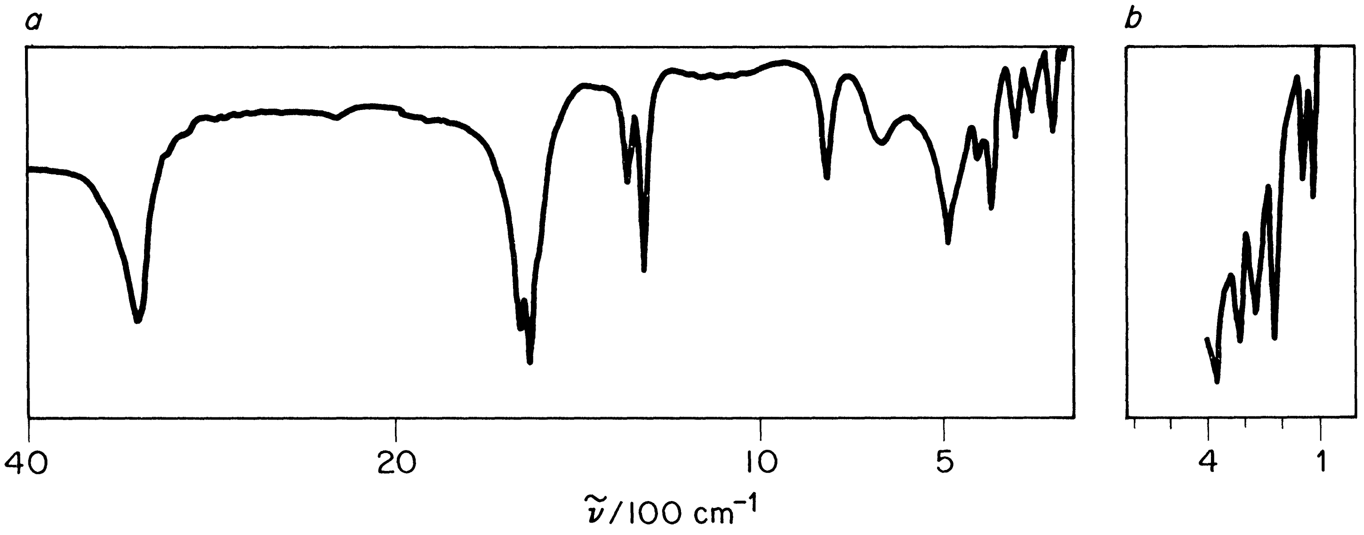

*Figure 3. Typical plotter output of spectra (synthetic magnesium oxalate) recorded from the 580B spectrometer (a) and the Fourier spectrometer (b). The overlapping spectral range of the two instruments is illustrated. The software automatically scales the abscissa axis to enable comparison of spectra from the two instruments.*

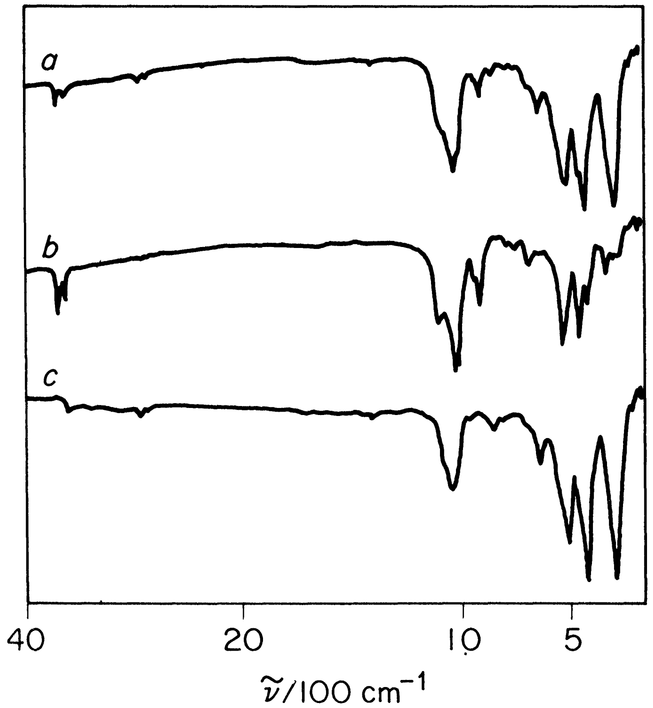

*Figure 4. An example of the use of the Manipulation module to generate the difference spectrum from two sample spectra. The spectrum of the parent clay material (b) is subtracted from a magnetically concentrated sample (a). The difference spectrum (c) is that characteristic of a new mineral, Macaulayite [12].*

graphics display on the monitor. The required plotting details and spectral file names are entered at the keyboard and the spectra retrieved from disc storage and displayed on the monitor as an aid in layout design. Eight spectra can be conveniently managed in one operation. Each spectrum can be moved on the graphics screen to optimize the plotting format, and scaling factors can be entered as required. The spectra in figure 4 illustrate the way in which three spectra can be manipulated to produce the required output design.

### *Terminal*

A machine-coded program allows the microcomputer to view the 580B spectrometer and the mainframe computer as intelligent terminals for commands and data transmission. Thus, data recorded in the infra-red laboratory can be formatted and processed before being passed to the larger computer for storage. It is anticipated that a comprehensive library of mineral and inorganic spectra may be produced in this way.

### *File development*

This is a modification of the Apple II FID routine and allows the operator to transfer data from files and between discs, to delete files, to rename files and produce printed catalogues of data discs.

### Summary

Integrated computers in molecular spectroscopy instrumentation first appeared with Fourier transform machines and this stimulated the progress in computerization of dispersive spectrometers [11]. With advances in microelectronics technology, and the increasing power and ease of use of microcomputers, it is to be expected that they will find more use in the analytical laboratory. Although the general-purpose microcomputer can provide an inexpensive alternative to the commercial, dedicated unit, the development of efficient software suitable for the untrained user can offset this cost-saving. The major advantage of the personal microcomputer is its flexibility—it is capable of application to a wide variety of interfacing and data-processing tasks. In the infra-red laboratory, the control of spectrometers by a separate microcomputer has permitted the development of extensive data manipulation programs in a high-level language. Updating this software, and the addition of new routines, is easily achieved because of the modular design of the programs. Compared with the dedicated microcomputer, this software can be relatively slow in operation. This is partly due to BASIC being translated during run-time, rather than compiled within the microcomputer. Machine-coded routines can provide high-speed operation and are frequently necessary for inter-instrument communication. The production of wholly machine-code programs would require extensive development time and would be unlikely to be cost-effective for most applications.

Further details of the interfacing and software described here can be obtained from the authors.

### Acknowledgements

The authors wish to express their thanks to Mr G. Gaskin for his aid in the interfacing of the Fourier spectrometer.

### References

1. Newton, D. A., *Laboratory Practice* (May 1982), 463.
2. Broadbridge, R. and Heslop, J. A., *International Laboratory* (April 1982), 72.
3. Anacreon, R. E. and Pattacini, S. C., *International Laboratory* (March 1980), 95.
4. Gluch, R. P., *International Laboratory* (June 1982), 80.
5. Coates, J. P., *Analytica Chimica Acta*, **103** (1978), 323.
6. Coates, J. P. and Geary, B., *Analytica Chimica Acta*, **103** (1978), 303.
7. Chipperfield, J. R. and Kirby, G. H., *Analytica Chimica Acta*, **132** (1981), 205.
8. Jones, D., *Practical Computing* (March 1980), 103.
9. Jones, R. N., *Applied Optics*, **8** (1969), 597.
10. Savitzky, A. and Golay, M. J. E., *Analytical Chemistry*, **36** (1964), 1627.
11. Steger, E. and Herzog, K., *Stud. Phys. Theor. Chem.*, **16** (1981), 259.
12. Wilson, M. J., Russell, J. D., Tait, J. M., Clark, D. R., Fraser, A. R. and Stephen, I., *Clay Minerals*, **16** (1981), 261.

